# Involvement of Ca^2+^ in Signaling Mechanisms Mediating Muscarinic Inhibition of M Currents in Sympathetic Neurons

**DOI:** 10.1007/s10571-022-01303-7

**Published:** 2022-11-11

**Authors:** Jin-Young Yoon, Won-Kyung Ho

**Affiliations:** 1 Department of Internal Medicine, Division of Cardiovascular Medicine, University of Iowa, 285 Newton Rd, 2283 CBRB, Iowa City, Iowa 52242 USA; 2 Department of Physiology, Seoul National University College of Medicine, Seoul, Korea; 3 Neuroscience Research Institute, Seoul National University College of Medicine, Seoul, Korea; 4 Department of Brain and Cognitive Science, Seoul National University College of Natural Science, Seoul, Korea

**Keywords:** KCNQ potassium channel, Muscarinic receptor, Ca^2+^, PIP_2_, PKC, Sympathetic neurons

## Abstract

Acetylcholine can excite neurons by suppressing M-type (KCNQ) potassium channels. This effect is mediated by M_1_ muscarinic receptors coupled to the G_q_ protein. Although PIP_2_ depletion and PKC activation have been strongly suggested to contribute to muscarinic inhibition of M currents (I_M_), direct evidence is lacking. We investigated the mechanism involved in muscarinic inhibition of I_M_ with Ca^2+^ measurement and electrophysiological studies in both neuronal (rat sympathetic neurons) and heterologous (HEK cells expressing KCNQ2/KCNQ3) preparations. We found that muscarinic inhibition of I_M_ was not blocked either by PIP_2_ or by calphostin C, a PKC inhibitor. We then examined whether muscarinic inhibition of I_M_ uses multiple signaling pathways by blocking both PIP_2_ depletion and PKC activation. This maneuver, however, did not block muscarinic inhibition of I_M_. Additionally, muscarinic inhibition of I_M_ was not prevented either by sequestering of G-protein βγ subunits from G_α_-transducin or anti-G_βγ_ antibody or by preventing intracellular trafficking of channel proteins with blebbistatin, a class-II myosin inhibitor. Finally, we re-examined the role of Ca^2+^ signals in muscarinic inhibition of I_M_. Ca^2+^ measurements showed that muscarinic stimulation increased intracellular Ca^2+^ and was comparable to the Ca^2+^ mobilizing effect of bradykinin. Accordingly, 20-mM of BAPTA significantly suppressed muscarinic inhibition of I_M_. In contrast, muscarinic inhibition of I_M_ was completely insensitive to 20-mM EGTA. Taken together, these data suggest a role of Ca^2+^ signaling in muscarinic modulation of I_M_. The differential effects of EGTA and BAPTA imply that Ca^2+^ microdomains or spatially local Ca^2+^ signals contribute to inhibition of I_M_.

**Supplementary Information:**

The online version contains supplementary material available at 10.1007/s10571-022-01303-7.

## Introduction

Neural M-type (KCNQ/Kv7) K^+^ channels are low threshold voltage-gated K^+^ channels that play a crucial role in regulating excitability of neurons (Delmas and Brown 2005; Cooper and Jan 2003). Breakdown of M channels by loss of function mutations or pharmacological inhibitors leads to neuronal hyperexcitability (Schroeder et al. 1998; Delmas and Brown 2005). Indeed, linopirdine, an M channel blocker, has acute cognition-enhancing properties in some animal experiments (Aiken et al. 1996), whereas M channel openers, such as retigabine, are currently being developed as novel antiepileptic drugs (Cooper and Jan 2003).

M currents (I_M_) were originally described in sympathetic neurons (Brown and Adams 1980; Constanti and Brown 1981) and named due to their inhibition by muscarinic acetylcholine receptors. However, the signal transduction mechanism underlying muscarinic inhibition of I_M_ remains uncertain. Despite the fact that muscarinic inhibition of I_M_ was shown to be mediated by G_q_-protein activation (Caulfield et al. 1994; Haley et al. 2000), typical downstream pathways of G_q_ such as PKC or IP_3_-mediated Ca^2+^ signals were not involved in muscarinic inhibition of I_M_ (Beech et al. 1991; Bosma and Hille 1989; Cruzblanca et al. 1998; del Rio et al. 1999). It was shown that neuronal I_M_ is encoded by KCNQ family genes (Wang et al. 1998) and activities of KCNQ channels depend on phosphatidylinositol 4,5-bisphosphate (PIP_2_) in the plasma membrane (Li et al. 2005; Suh et al. 2006; Zhang et al. 2003; Kim et al. 2016). Recent progress elucidated that PIP_2_ modulate KCNQ2/KCNQ3 channel opening by interacting synergistically with a minimum of four cytoplasmic domains (Hernandez et al. 2008a; Choveau et al. 2018). These led to a hypothesis that depletion of PIP_2_ by muscarinic stimulation is responsible for inhibition of I_M_ (Delmas and Brown 2005; Suh and Hille 2002).

I_M_ is negatively modulated by other G_q_-coupled neurotransmitter receptors, such as bradykinin B_2_ receptors, and purinergic P2Y receptors (Bofill-Cardona et al. 2000; Jones et al. 1995). The mechanism involved in bradykinin-induced inhibition of I_M_ has been particularly well characterized, and it has been shown that calmodulin activation induced by IP_3_-mediated intracellular Ca^2+^ signals is responsible for I_M_ inhibition (Gamper et al. 2005; Gamper and Shapiro 2003). At present, it appears to be generally accepted that I_M_ is regulated by three modulatory pathways: PIP_2_ depletion for muscarinic inhibition and second messengers, such as Ca^2+^/CaM for bradykinin-induced inhibition (Hernandez et al. 2008b), protein kinase C (Lee et al. 2010; Kosenko et al. 2012), and post-translational modification (Kim et al. 2016; Qi et al. 2014). However, despite many studies investigating the PIP_2_ depletion hypothesis for the mechanism of muscarinic inhibition of I_M_ (Ford et al. 2003; Suh and Hille 2002; Suh et al. 2006; Winks et al. 2005; Zhang et al. 2003), direct evidence is still lacking. In addition, since PIP_2_ mobility determines the spatial and temporal profiles of PIP_2_ depletion, the idea that PIP_2_ depletion acts as the M_1_ muscarinic signal in neurons requires that PIP_2_ diffusion be slow (Cho et al. 2005). Yet, in general, PIP_2_ diffuses rapidly in neurons (van Rheenen and Jalink 2002; Cho et al. 2005), making this hypothesis unlikely. The PIP_2_ depletion hypothesis was supported by a result demonstrating that increasing PIP_2_ concentration of sympathetic neurons by overexpressing the synthetic enzyme phosphoinositide-5 (PI-5)-kinase can block muscarinic inhibition of I_M_. Kruse and Whitten recently developed a model that describes altering M_1_R surface density and PI-5-kinase activity regulating the excitability of rat SCG neurons (Kruse and Whitten 2021). However, it is not certain whether increased resting PIP_2_ levels caused by overexpression of PI-5-kinase can inhibit PIP_2_ depletion induced by receptor-mediated activation of PLC (Winks et al. 2005). Given that PIP_2_ can modulate a variety of cellular processes, including cortical actin organization, membrane ruffling, vesicle trafficking, and gene expression (Di Paolo and De Camilli 2006), the possibility that normal signaling mechanisms involved in muscarinic inhibition of I_M_ are altered in excess PIP_2_ cannot be excluded. To prove whether receptor-mediated inhibition is mediated by PIP_2_ depletion, it is important to demonstrate that the inhibition is rescued by applying normal concentrations of exogenous PIP_2_. Indeed, inhibition of GIRK channels by phenylephrine, endothelin, and PGF2α in cardiac myocytes, which were shown to be mediated via PIP_2_ depletion, are completely abolished by internally perfused 10-μM PIP_2_ (Cho et al. 2005, 2001). However, muscarinic inhibition of KCNQ channels was only partially reduced even by 500-μM PIP_2_ (Robbins et al. 2006), which can be considered inconsistent with the PIP_2_ depletion hypothesis. In the present study, we investigated the mechanism involved in muscarinic inhibition of I_M_ by examining various possible signaling components in rat superior cervical ganglion (SCG) sympathetic neurons and HEK cells expressing KCNQ channels.

## Materials and Methods

### Ethics Approval

This study was reviewed and carried out in accordance with the Institutional Animal Care and Use Committee (IACUC) at Sungkyunkwan University School of Medicine (SUSM). SUSM is an Association for Assessment and Accreditation of Laboratory Animal Care International accredited facility and abides by the Institute of Laboratory Animal Resources Guide. The animals were maintained in standard environmental conditions (25 ± 2 °C; 12/12-h dark/light cycle), were given ad libitum access to water and food, and were housed under veterinary supervision at the Laboratory Animal Research Center, SUSM. Sprague–Dawley rats were purchased from Orient Bio Inc. (Sungnam, South Korea). The authors understand the ethical principles under which The Journal of Physiology operates and the experiments comply with the animal ethics checklist described in Grundy (Grundy 2015).

### Cell Culture, Transfection, and SCG Isolation

SCG neurons were cultured from 3- to 4-week-old male rats as previously described (Gamper et al. 2004). HEK293 cells were handled as previously described (Cho et al. 2006). Transfections were made using Lipofectamine 2000 reagents (Invitrogen, Carlsbad, CA) and green fluorescent protein (GFP) was used as a reporter. Plasmids encoding human KCNQ2 (GenBank accession number AF110020) and rat KCNQ3 (GenBank accession number AF091247) were kindly provided by Mark Shapiro (University of Texas Health Science Center, San Antonio, TX).

### Electrophysiological Recordings

Current measurements were made with the whole-cell patch-clamp technique. Voltage clamp was performed with an EPC-8 amplifier (HEKA Instruments, Lambrecht, Germany). Filtered signals (1–2 kHz) from a patch-clamp amplifier were fed into an AD/DA converter (PCI-MIO-16E-4, National Instruments, Austin, TX), digitized at 5 kHz and stored digitally in later analysis. Electrodes were pulled from borosilicate capillaries (World Precision Instruments, Inc., Sarasota, FL) using a pipette puller (PP-83, Narishige, Tokyo) and positioned precisely with a micromanipulator (MP 225, Sutter Instrument Company, Novato, CA). The pipettes had a resistance of 2–3 MΩ when filled with pipette solution. Data were not corrected for liquid junction potential (−9 mV). For dialysis, we waited > 5 min before starting the experiment. The perfusion system was a homemade 100-µl perfusion chamber through which solution flowed continuously at 5 ml/min. All recordings were carried out at room temperature (22–24 °C).

The normal external solution for HEK293 cell and rat SCG neuron recording was as follows (in mM): 143 NaCl, 5.4 KCl, 5 HEPES, 0.5 NaH_2_PO_4_, 11.1 glucose, 0.5 MgCl_2_, 1.8 CaCl_2_, and pH 7.4 adjusted with NaOH. The pipette solution was as follows (in mM): 126 KMeSO_4_, 14 KCl, 10 HEPES, and 3 MgCl_2_ (pH 7.24 adjusted with KOH). 500 nM of TTX (Tocris, St. Louis, MO, Cat. #1069) and 0.1 μM of CdCl_2_ were included in external solution to block Na^+^ and Ca^2+^ currents.

### [Ca^2+^]_i_ Measurement

[Ca^2+^]_I_, the concentration of intracellular free Ca^2+^, was measured by loading cells with 2-μM Fura 2-acetoxymethyl ester (Fura 2-AM) (Molecular Probes, Eugene, OR) for 30 min in the recording medium. Changes in Ca^2+^ were estimated from the ratio of Fura 2 fluorescence (at 500 nm) with alternate excitation at wavelength of 340 and 380 nm, generated by a Lambda DG-4 monochromator (Sutter Instrument Company). Fura 2 ratio data (F340/380), [Ca^2+^]_i_, were measured at a frequency of 2 Hz. Intensity of illumination was minimized to reduce bleaching of Fura 2, while maintaining adequate levels of signal. Fura 2 images for digital analysis were generated using a system based on an Olympus IX71 microscope coupled to an image intensifier and CCD camera and were analyzed with MetaFluor software (MDS Analytical Technologies, Sunnyvale, CA).

### Statistical Analysis

Results in the text and the figures are presented as mean ± S.E.M. (n = number of cells tested). Statistical analyses were performed using Student’s *t* test. *P*‐values of < 0.05 were considered statistically significant.

## Results

### Characterization of I_M_ and Muscarinic Modulation of M Channels in SCG Neurons

**Fig. 1:**
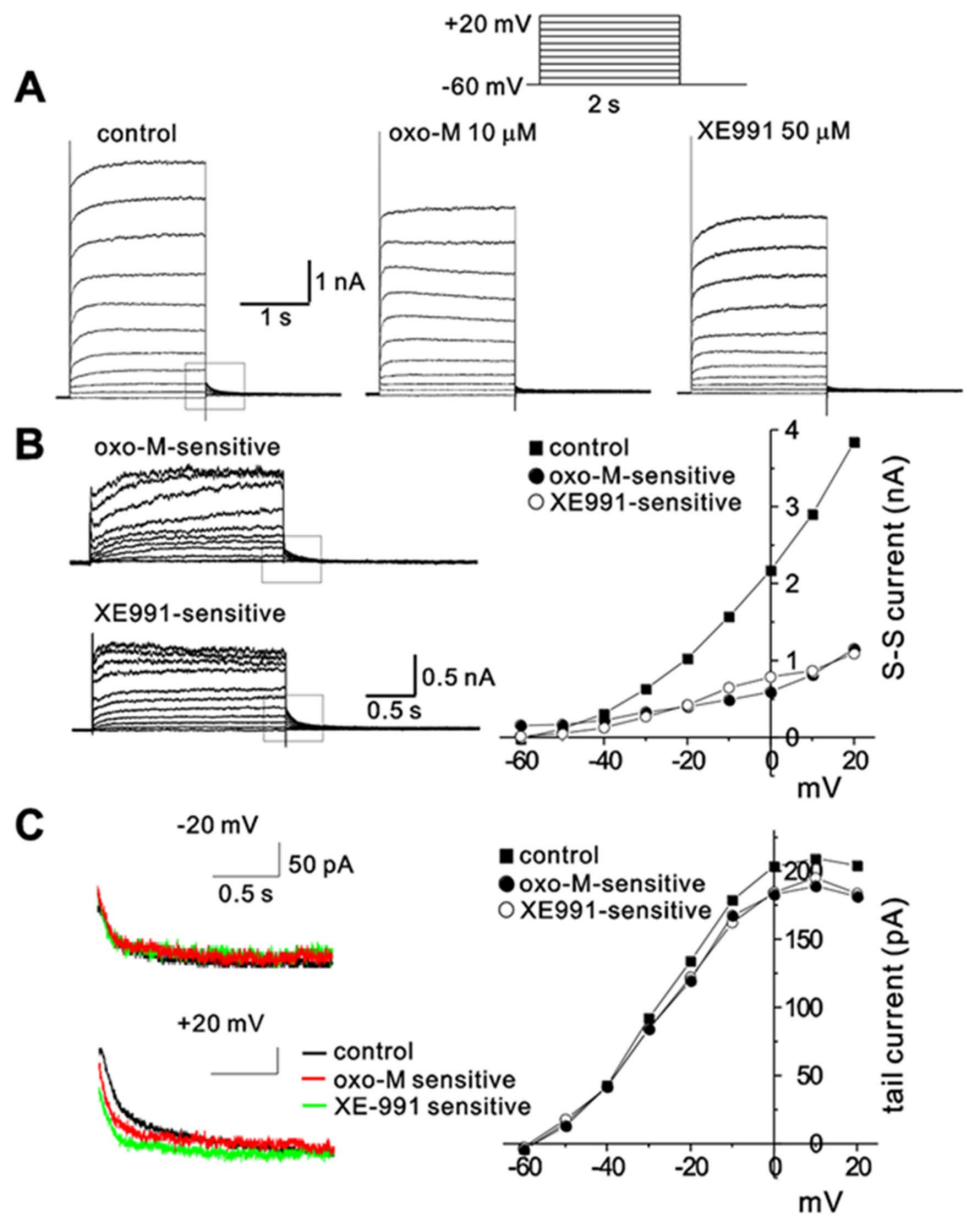
Isolation of I_M_ from SCG neurons. **A** Families of current elicited by voltage steps from −60 mV to + 20 mV, in 10-mV intervals, before (left panel) and after application of 10-μM oxo-M (middle) or 50-μM XE991 (right). Holding potential −60 mV. *Inset* shows the pulse protocol. **B** *left*, oxo-M-sensitive and XE991-sensitive currents; *right,* steady-state current–voltage (*I–V*) relationships for oxo-M-sensitive and XE991-sensitive currents shown in *left*. **C** *left*, superimposed deactivation tails for control (black), oxo-M sensitive (red), and XE991-sensitive currents (green) by hyperpolarizing steps to −60 mV from −20 mV (upper panel) or + 20 mV (lower panel); *right, I–V* relationships for control, oxo-M-sensitive, and XE991-sensitive tail currents

I_M_ was originally characterized as outward K^+^ currents suppressed by muscarinic stimulation (Brown and Adams 1980) and specific blockers to inhibit I_M_, such as linoperdine and XE991, were developed later (Aiken et al. 1996; Cooper and Jan 2003). To investigate the mechanism of muscarinic modulation of I_M_ in rat SCG neurons, we first compared the current component inhibited by oxotremorine-M (oxo-M), a muscarinic agonist, and that inhibited by XE991. Here we used the whole-cell patch-clamp mode, a method suitable for preserving intracellular microdomains, investigating localized signaling, and directly modulating the signaling molecules driving muscarinic inhibition of M channels. To prevent the potential “run-down” of I_M_ in the whole-cell configuration, after the pipette ruptured the membrane, we allowed I_M_ to stabilize before tracking muscarinic inhibition. Step depolarizing pulses were applied in 10-mV steps from a holding potential of −60 mV. In Fig. 1A, representative current traces obtained in the control condition and after applying oxo-M (10 μM) or XE991 (50 μM) are shown. We obtained the current component inhibited by oxo-M (oxo-M-sensitive) and that inhibited by XE991 (XE991 sensitive) by subtracting current traces in the presence of oxo-M or XE991, respectively, from the control current traces (Fig. 1B, left panel). In Fig. 1B (*right*), current–voltage (*I-V*) relationships for total currents in control (squares), oxo-M-sensitive currents (closed circles), and XE-991-sensitive currents (open circles) were plotted. The plot shows that oxo-M-sensitive currents and XE-991-sensitive currents are similar over the voltage range tested between −60 mV and + 20 mV. Assuming that XE-991-sensitive currents represent I_M_, I_M_ comprises 26.49 ± 1.41% of total outward currents at + 20 mV and 10-μM oxo-M inhibits I_M_ by 79.90 ± 13.62% (at + 20 mV, *n* = 7).

In Fig. 1C, we compared deactivation tail currents (I_tail_) recorded upon repolarization (indicated as square boxes in Figs. 1A and B) and plotted the amplitude of I_tail_ against step depolarization voltage. To avoid contamination of capacitive currents and fast components that may not be attributable to I_M_, we measured the amplitude of I_tail_ by measuring the average current level between 30 and 50 ms after repolarization and subtracting the current level at steady state. The result shows the similarity between I_tail_ of oxo-M-sensitive currents and XE-991-sensitive currents. Furthermore, at voltage ranges up to −20 mV, I_tail_ of oxo-M-sensitive currents and XE-991-sensitive currents are almost equivalent to I_tail_ of control currents, indicating that I_tail_ corresponding to −20-mV depolarization is mostly attributable to I_M_. In the present study, therefore, we regarded I_tail_ as an indication of I_M_.

**Fig. 2:**
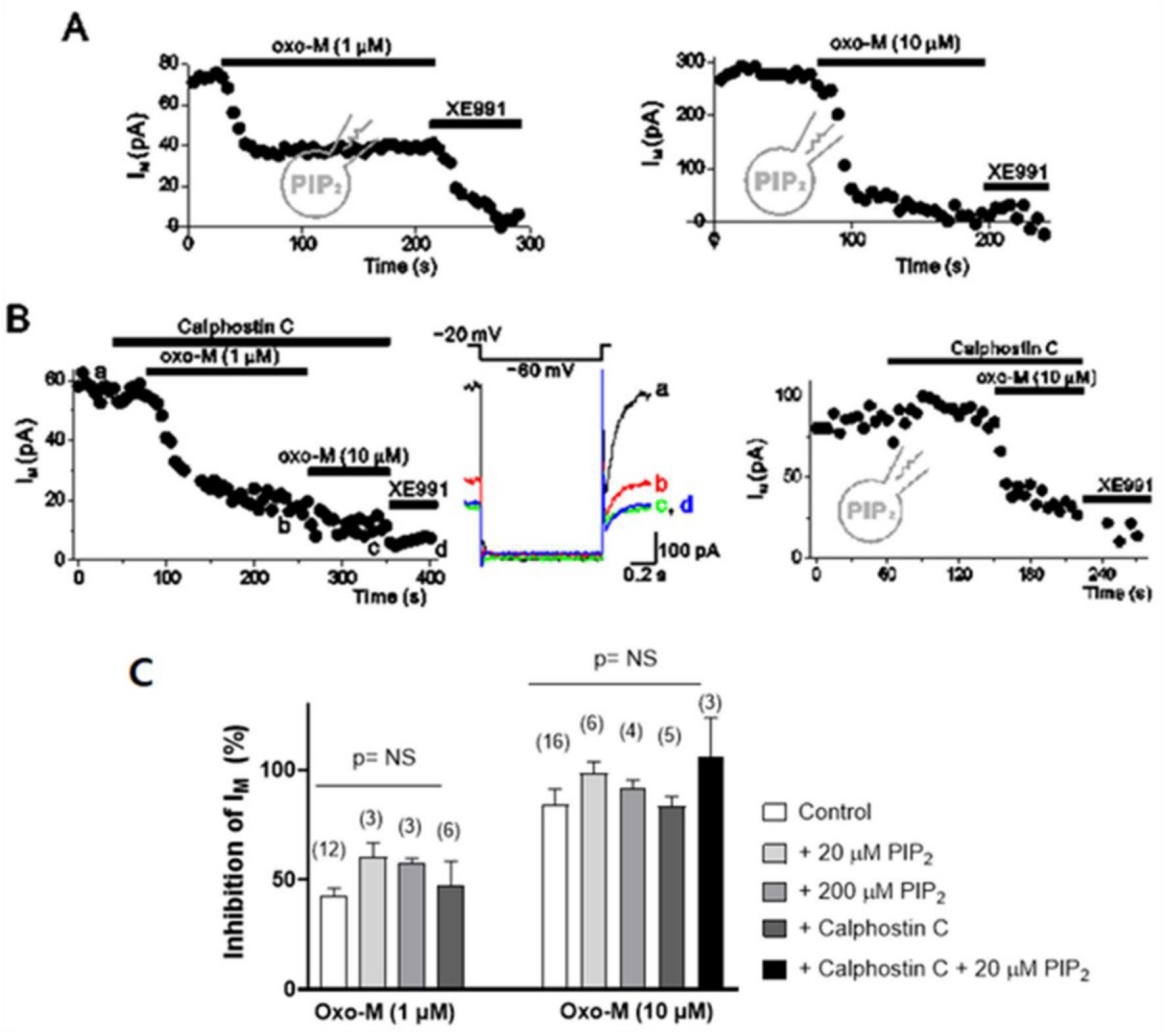
Muscarinic inhibition of I_M_ was not blocked either by supply of PIP_2_ or by PKC inhibitor. The current amplitudes were measured as the deactivation tail current induced by 1-s hyperpolarizing steps to −60 mV from a holding potential of −20 mV at 5-s intervals. **A** The effects of 1-µM oxo-M (*left*) or 10-μM oxo-M (*right*) on I_M_ in cells which were patched with 20-μM diC8-PIP_2_. **B** Time course of I_M_ amplitude during cumulatively increasing concentration of oxo-M indicated. Neurons were pretreated by 1-µM calphostin C without (*left*) and with (*right*) 20-µM diC8-PIP_2_ in the patch pipet. *Inset* shows the pulse protocol and representative current traces of *left* panel. **C** Summary of the percent inhibitions of I_M_ by oxo-M in control conditions or in cells applied with various concentrations of PIP_2_ or calphostin C. Values are expressed as means ± S.E.M. NS, not significant (*p* > 0.05)

The concentration-dependent effects of oxo-M to inhibit I_M_ were tested, while changes of I_tail_ were monitored in 5-s intervals by applying a hyperpolarizing pulse to −60 (or −55) mV for 1.5 s from the holding potential of -20 mV. Extent of inhibition induced by 1-μM and 10-μM oxo-M were 42.54 ± 3.51% (n = 12) and 84.13 ± 7.34% (n = 16), respectively (Fig. 2C).

### Re-examination of the Current Hypothesis: PIP_2_ Depletion and PKC Activation

PIP_2_ depletion and PKC activation have been considered to be the most likely candidates involved in muscarinic inhibition of I_M_ (Delmas and Brown 2005). However, previous studies have found that muscarinic inhibition of I_M_ was not fully blocked by application of exogenous PIP_2_ or PKC inhibitors (Brown and Yu 2000; Ford et al. 2003; Hoshi et al. 2003; Robbins et al. 2006; Shapiro et al. 2000). We confirmed that exogenous PIP_2_ (up to 200 μM) had little effect on muscarinic inhibition of I_M_ (Fig. 2A). Consistent with the data from sympathetic neurons, in HEK293 cells expressing KCNQ2/KCNQ3 channels and M1 muscarinic receptors, application of PIP_2_ did not block the effect of oxo-M on I_M_ (Yoon 2010). To apply PIP_2_ into cells, diC8-PIP_2_ was added to the pipette solution, and the conventional whole-cell mode was accomplished by rupturing membranes after a giga-seal was made. We have recently shown that application of diC8-PIP_2_ disrupts M channel regulation induced by the altered channel’s affinity for PIP_2_ (Lee et al. 2010), suggesting that exogenous PIP_2_ is effectively delivered to the plasma membrane and available to M channels. We also confirmed that calphostin C, a highly specific PKC inhibitor, did not affect muscarinic inhibition of I_M_ (Fig. 2B). Summarized data are shown in Fig. 2C. In addition, a higher dose of calphostin C (up to 10 μM) and two other PKC inhibitors, 1-μM chelerythrine and 100-nM bisindolylmaleimide I, were tested and neither affected oxo-M-induced M current modulation (SI 1).

We then hypothesized that muscarinic inhibition of I_M_ uses multiple signaling pathways such that inhibition can only be blocked when multiple pathways are simultaneously suppressed. To test this hypothesis, both PIP_2_ depletion and PKC activation were prevented with exogenous PIP_2_ (20 μM) and calphostin C (1 μM). In the presence of 20-μM PIP_2_ and calphostin C, however, 10-μM oxo-M was still capable of inhibiting I_M_ (Fig. 2B and C).

### Looking for New Possibilities

**Fig. 3:**
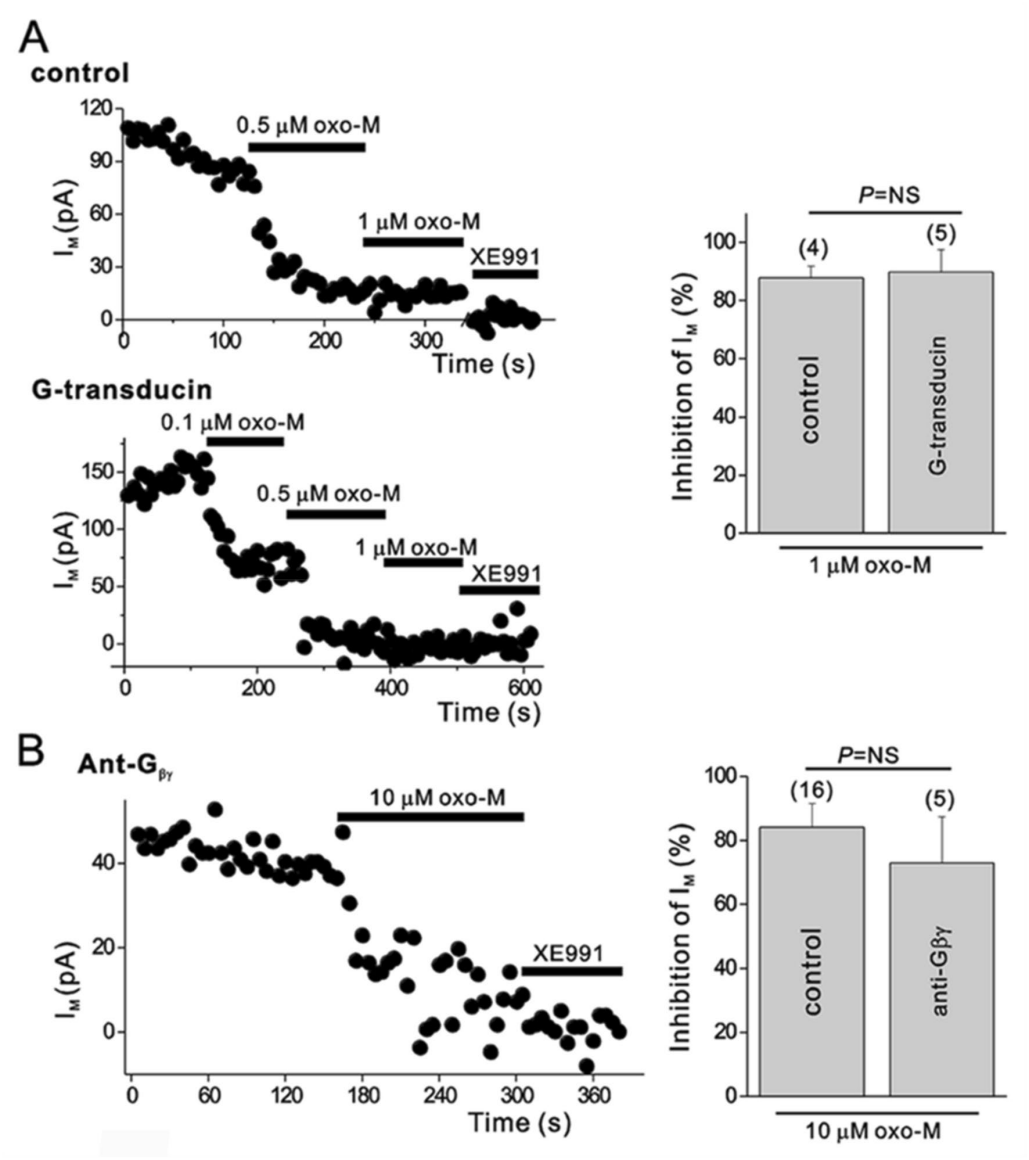
G_βγ_-buffering proteins did not affect muscarinic modulation of I_M_. **A** *Left*, time course of I_M_ amplitude during cumulatively increasing concentrations of oxo-M in control HEK cells expressing KCNQ2/3 channels and M_1_ muscarinic receptors (upper panel) and cells expressing channels and M1Rs plus ﻿Gα-transducin (lower panel). *Right*, summary of the percent inhibitions of I_M_ by oxo-M, collected as in *left*. **B** *Left*, time course of I_M_ in SCG neurons loaded with anti-G_βγ_ antibody. *Right*, summarized data for the effects of anti-G_βγ_ antibody on muscarinic inhibition of I_M_. Values are expressed as means ± S.E.M

Since we did not find evidence that messengers downstream of the Gα_q_ pathway mediate muscarinic inhibition of I_M_, we searched for other possibilities. It is well known that the G_βγ_ dimer mediates voltage-dependent modulation of N-type Ca^2+^ channels through numerous neurotransmitters, including noradrenaline, acetylcholine, and dynorphin (Herlitze et al. 1996; Ikeda 1996; Tsien et al. 1988). We tested the involvement of the G_βγ﻿_ subunit in oxo-M-induced I_M_ modulation using HEK cells transfected with G_α_-transducin (G_α_TD) together with KCNQ2, KCNQ3, and M_1_ muscarinic receptors. G_α_TD is a G_α_ subunit which predominantly attains the GDP-bound state when overexpressed in SCG neurons and buffers G_βγ﻿_ (Kammermeier and Ikeda 1999). As shown in Fig. 3A, expression of G_α_TD did not affect muscarinic inhibition of the KCNQ current by 1-μM oxo-M (87.84 ± 4.02% (*n*= 4) in controls or 89.77 ± 7.74% (*n* = 5) in G_α_TD-expressed cells, *p* > 0.05). In addition, scavenging G_βγ﻿_ subunits with intracellular anti-G_βγ﻿_ antibody in SCG neurons was also tested. In the presence of anti-G_βγ﻿_ antibody, 10-μM oxo-M-induced inhibition of M current was not different from control conditions. (Fig. 3B). These data implicate that G_βγ﻿_ subunits do not have a critical role in muscarinic inhibition of I_M_.

Recently, it was reported that receptor activation regulates channel activity by affecting the surface expression of channel proteins (Chung et al. 2009). Because KCNQ channels are known to be targets of trafficking, i.e., insertion into and removal from the plasma membrane (Etxeberria et al. 2008; Schuetz et al. 2008), we tested the possibility that KCNQ channel trafficking is involved in muscarinic inhibition of I_M_. However, a block of protein trafficking with 50-μM blebbistatin, a cell-permeable inhibitor of class-II myosins, did not affect muscarinic inhibition of I_M_ (SI 2). In addition, pretreatment of a protein trafficking inhibitory cocktail including chloroquin (100 μM), monensin (100 μM), and nocodazole (20 μM) had no effect on muscarinic modulation of I_M_ (data not shown), suggesting that KCNQ channel trafficking did not contribute to the regulation of I_M_ by M_1_ muscarinic receptors.

### Revisiting Ca^2+^-Dependent Hypothesis

Recently, the role of Ca^2+^ signaling in I_M_ inhibition was well established for I_M_ inhibition by bradykinin (Gamper et al. 2005; Gamper and Shapiro 2003) or by P2Y receptor stimulation (Zaika et al. 2007), in which CaM was shown to be responsible. However, inhibition of CaM activation using dominant-negative CaM that cannot bind Ca^2+^ (Geiser et al. 1991) did not affect muscarinic inhibition of I_M_ (Gamper and Shapiro 2003; Zaika et al. 2007), leading to the conclusion that Ca^2+^ signaling is not involved in muscarinic inhibition of I_M_. This conclusion was supported by the observation that in SCG neurons, oxo-M did not induce Ca^2+^ transients as robustly as bradykinin did (Cruzblanca et al. 1998; del Rio et al. 1999). In early experiments, however, muscarinic inhibition of I_M_ was shown to be suppressed when intracellular Ca^2+^ was heavily buffered (Beech et al. 1991). Further, Shapiro et al. (Shapiro et al. 2000) reported that oxo-M-induced Ca^2+^ transients are blocked by thapsigargin. Taken together, these findings implicate Ca^2+^ signaling in muscarinic receptor stimulation. Thus, we re-examined the roles of Ca^2+^ signaling in muscarinic inhibition of I_M_ inhibition.

**Fig. 4:**
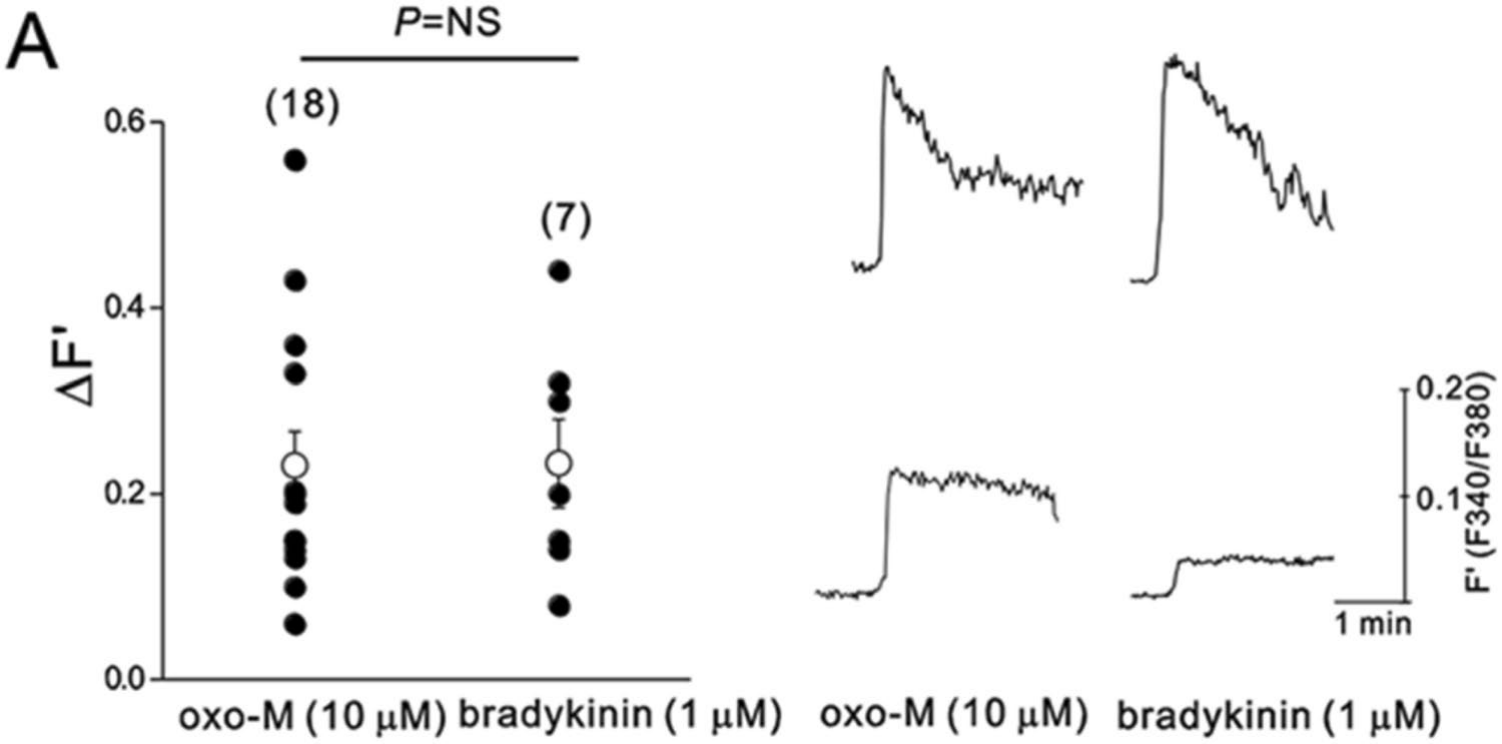
Muscarinic stimulation raises [Ca^2+^]_i_ in rat SCG neurons. SCG neurons were loaded with 2-μM Fura 2-AM for Ca^2+^ measurements. The F340/F380 ratio was used to estimate [Ca^2+^]_*i*_. F340/F380 ratio (F´) changes in oxo-M or bradykinin-stimulated cells was summarized. Open circles indicate means ± S.E.M. *Inset* shows two representative [Ca^2+^]_i_ recordings of each group

First, we examined whether oxo-M could induce Ca^2+^ transients in SCG neurons and whether oxo-M-induced Ca^2+^ transients would be significantly smaller than bradykinin-induced Ca^2+^ transients, as previously described (Cruzblanca et al. 1998; del Rio et al. 1999). [Ca^2+^]_i_ was measured in SCG neurons using Fura 2-AM. As shown in Fig. 4, 10-μM oxo-M did induce Ca^2+^ transients in all SCG neurons tested though there was large cell-to-cell variation. On average, oxo-M-induced Ca^2+^ transients were not different from bradykinin-induced Ca^2+^ transients (Fig. 4). The peak amplitude of Ca^2+^ transient induced by oxo-M was similar to that induced by bradykinin. Paired *t* test confirmed that the effects of oxo-M on intracellular Ca^2+^ concentration were not significantly different from those of bradykinin in the same neurons (*n* = 3, *p > *0.05). These results imply that the Ca^2+^-releasing powers of oxo-M and bradykinin are not significantly different.

**Fig. 5:**
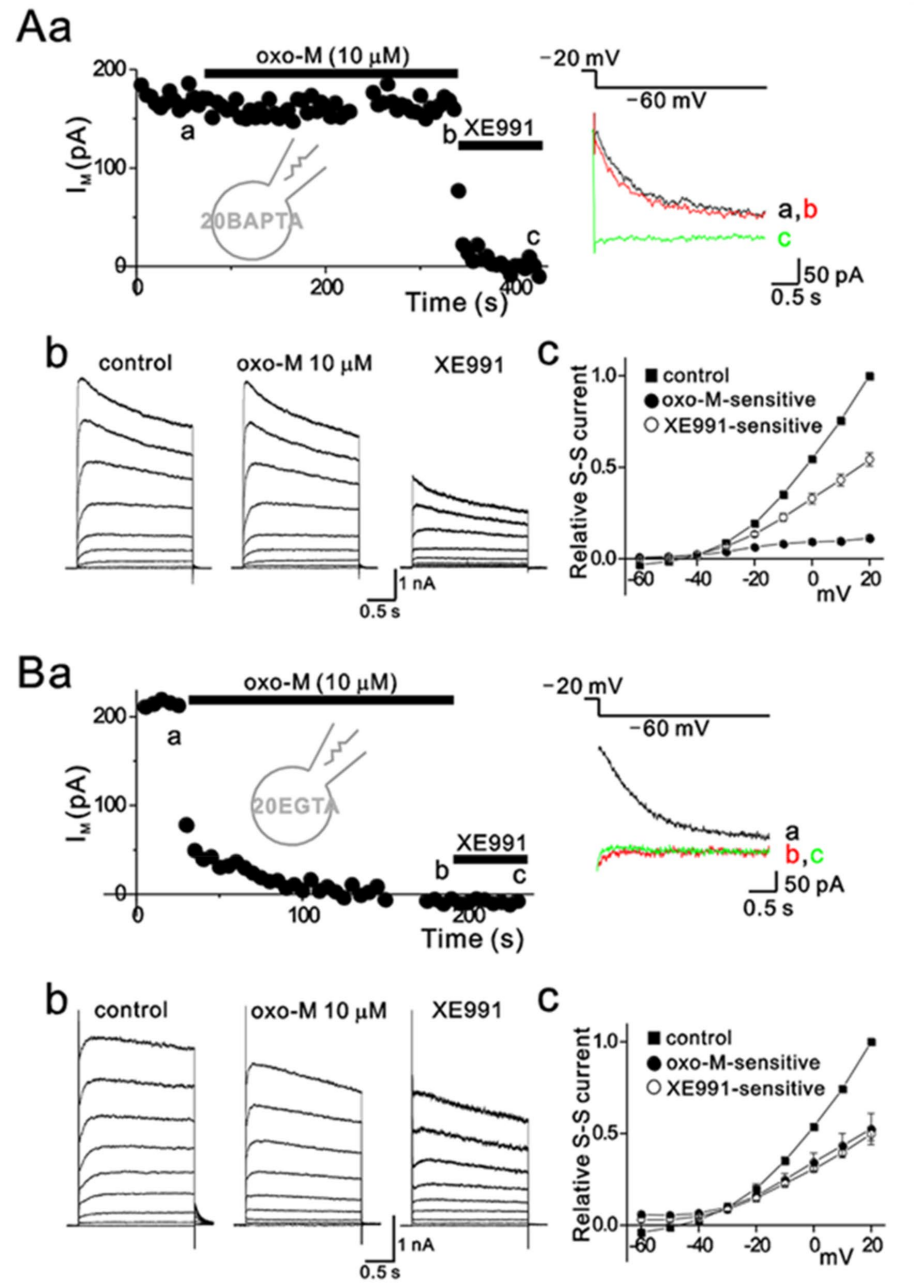
Intracellular BAPTA, but not EGTA suppresses muscarinic modulation of I_M_. The current inhibition induced by 10-μM oxo-M were measured in cells dialyzed with 20-mM BAPTA (**A**) or 20-mM EGTA (**B**). *a*, time course of tail current amplitude induced by the pulse protocol as shown in the inset. *Right*, superimposed deactivation tail currents before (black) or after application of oxo-M or XE991. *b*, families of current elicited by voltage steps from −60 mV to + 20 mV, in 10-mV intervals, before (left panel) and after application of 10-μM oxo-M (middle panel) or XE991 (right panel) in a cell shown in *a*. *c*, pooled data for normalized oxo-M-sensitive (closed circles) and XE991-sensitive steady currents (open circles) to control steady-state currents at + 20 mV, collected as in *b*

Since muscarinic receptor stimulation elicited an increase of [Ca^2+^]_i_, we tested whether Ca^2+^ mediates muscarinic modulation of I_M_ using 20-mM BAPTA. When the pipette solution contained 20-mM BAPTA, oxo-M minimally affected I_M_ (Fig. 5Aa). Superimposed deactivation tail currents showed that in a cell dialyzed with 20-mM BAPTA, I_M_ after 5 min oxo-M treatment was almost overlapped with that in controls. To rule out the possibility that I_M_ itself was reduced by 20-mM BAPTA, we obtained the steady-state current–voltage relationships for oxo-M-sensitive currents and XE-991-sensitive currents in BAPTA-loaded cells, using the same protocol as in Fig. 1. Figure 5Ac demonstrates the proportion of XE991-sensitive currents to total steady-state currents in 20-mM BAPTA-loaded cells was not reduced compared to that in 0.1-mM EGTA (52.40 ± 2.46% [*n* = 19] *vs.* control: 26.49 ± 1.41% [*n* = 7] at + 20 mV). The oxo-M-induced inhibition of I_M_ was determined as a proportion of oxo-M-sensitive currents to XE991-sensitive currents at + 20 mV as in Fig. 1 and was 22.21 ± 3.94% (*n* = 19, Fig. 5Ac), which was significantly smaller than that in controls (79.90 ± 13.62% (*n* = 7), *p* < 0.01). We then tested whether the effect of BAPTA on suppressing muscarinic inhibition of I_M_ could be attributed to inhibiting local Ca^2+^ signals. To do this, we tested the effect of EGTA on muscarinic inhibition of I_M_. BAPTA is a fast Ca^2+^ buffer that effectively buffers all types of Ca^2+^ signals, whereas EGTA is a slow Ca^2+^ buffer that selectively spares local Ca^2+^ signals (Neher 1998). With 20-mM EGTA, muscarinic receptors were still able to inhibit M currents (Fig. 5Ba). The oxo-M-induced inhibition of I_M_ was calculated as a proportion of oxo-M-sensitive currents to XE991-sensitive currents at + 20 mV and was 103.05 ± 11.60% (*n* = 5, Fig. 5Bc), which was not significantly different from that of control conditions (*p* > 0.05). We confirmed that proportions of I_M_ to total outward currents in the presence of 20-mM EGTA (49.60 ± 3.48% [*n* = 4]) at + 20 mV) were similar to those in the presence of 20-mM BAPTA (52.40 ± 2.46% [*n* = 19]).

Next, we directly examined the effect of [Ca^2+^]_i_ on M currents in SCG neurons. We perfused cells with a bathing solution containing 10-μM ionomycin, a Ca^2+^ ionophore, and 10-mM CaCl_2_ to raise [Ca^2+^]_i_. We found that as [Ca^2+^]_i_ increased, the M current amplitude declined in parallel (SI 3). This is consistent with previous data showing the sensitivity of KCNQ2/KCNQ3 channels to [Ca^2+^]_i_ (Delmas and Brown 2005; Gamper and Shapiro 2003; Kosenko and Hoshi 2013). Taken together, these data suggest that Ca^2+^ signals play a critical role in M_1_ muscarinic receptor-mediated regulation of M channels. The differential effects of EGTA and BAPTA imply that M channels are very close to Ca^2+^ sources and are regulated by local Ca^2+^ signals.

## Discussion

We have demonstrated that muscarinic inhibition of I_M_ in rat SCG neurons is significantly suppressed by 20-mM BAPTA in pipette solutions (Fig. 5). In fact, similar findings were observed in earlier studies (Beech et al. 1991; Kirkwood et al. 1991). However, Ca^2+^ was not regarded as a candidate for couple muscarinic receptor activation to M channel inhibition, because oxo-M failed to increase the intracellular Ca^2+^ concentration in SCG neurons (Beech et al. 1991; del Rio et al. 1999). The current understanding of the distinction between muscarinic receptor-induced inhibition of I_M_ and bradykinin-induced inhibition of I_M_ relies, at least in part, on the inability of oxo-M to induce Ca^2+^ increase (Delmas and Brown 2005). However, some controversial reports stated that oxo-M induces Ca^2+^ increase in acutely dissociated rat SCG neurons (del Rio et al. 1999; Cruzblanca et al. 1998) and tsA-201 cells (Falkenburger et al. 2013). Furthermore, oxo-M was invariably shown to induce Ca^2+^ mobilization in other parts of the brain (Irving and Collingridge 1998; Seymour-Laurent and Barish 1995), and oxo-M-induced effects, such as GIRK channel inhibition, were shown to be mediated by Ca^2+^-dependent mechanisms (Sohn et al. 2007). Other reports exploring the PIP_2_ depletion hypothesis showed that M current could be inhibited by muscarinic receptors in neurons overexpressing the neuronal calcium sensor, NCS-1 (Winks et al. 2005). NCS-1 up-regulates PI-4-kinase, which catalyzes the first step in PIP_2_ synthesis from PI and requires Ca^2+^ to be activated (Koizumi et al. 2002; Rajebhosale et al. 2003; Zhao et al. 2001; Taverna et al. 2002; Burgoyne and Weiss 2001). In neurons overexpressing NCS-1, Winks et al. reported that M current inhibition by oxo-M was conserved, while the inhibitory effect of bradykinin was reduced (Winks et al. 2005). They claimed that oxo-M does not evoke enough Ca^2+^ release to activate NCS-1 for replenishing PIP_2_ hydrolyzed by muscarinic stimulation. However, others reported similar basal Ca^2+^ levels, ~ 100 nM, in PC12 cells and SCG neurons, which are enough to activate NCS-1 in PC12 cells (Cruzblanca et al. 1998; Delmas et al. 2002; Meijer et al. 2014; Koizumi et al. 2002; Rajebhosale et al. 2003; Taverna et al. 2002). In this case, under basal conditions during muscarinic receptor activation, NCS-1 could compensate for PIP_2_ consumption by PLC. If PIP_2_ depletion hypothesis is true, in cells overexpressing NCS-1, oxo-M is unlikely to inhibit M current because NCS-1 replenishes PIP_2_ but supporting our hypothesis that PIP_2_ depletion is not the primary mechanism.

In the present study, we re-examined whether the inability of oxo-M to induce Ca^2+^ mobilization is a consistent finding. Surprisingly, we found that muscarinic receptor stimulation by oxo-M is capable of increasing intracellular Ca^2+^ concentration in rat sympathetic neurons. We noted that the cell-to-cell variation of the amplitude of Ca^2+^ transients was quite large; thus, in some cells, Ca^2+^ transients induced by oxo-M were small, which was similar to a previous report (Delmas and Brown 2002). However, this was also the case for B2R-induced Ca^2+^ transients (Fig. 4). Thus, we conclude that the current understanding of dual modulatory pathways for M channel regulation based on different Ca^2+^ mobilization powers, the ability to evoke changes of [Ca^2+^]_i_, needs to be re-examined. We do not know the reason for the discrepancy of Ca^2+^ mobilization power of oxo-M among different studies, but differences in receptor density (Dickson et al. 2013; Falkenburger et al. 2013; Kruse and Whitten 2021), signaling microdomains that modify the efficacy of IP_3_ to open its receptor (Zaika et al. 2011), or experimental conditions may be involved. Previous studies used voltage-clamped neurons with Fura 2 in pipette solutions and held the membrane potential near or below −60 mV during Ca^2+^ measurements (Beech et al. 1991; del Rio et al. 1999; Falkenburger et al. 2013; Dickson et al. 2013), whereas we used intact sympathetic neurons with Fura 2-AM. If Ca^2+^ entry through the voltage-dependent ion channels contributed to the oxo-M-induced [Ca^2+^]_i_ rises, this pathway might have been blocked by clamping the membrane potential hyperpolarized to −60 mV or below. The fact that removal of external Ca^2+^ prevented the rise of [Ca^2+^]_i_ by muscarinic receptor stimulation may support this possibility (Foucart et al. 1995).

**Fig. 6:**
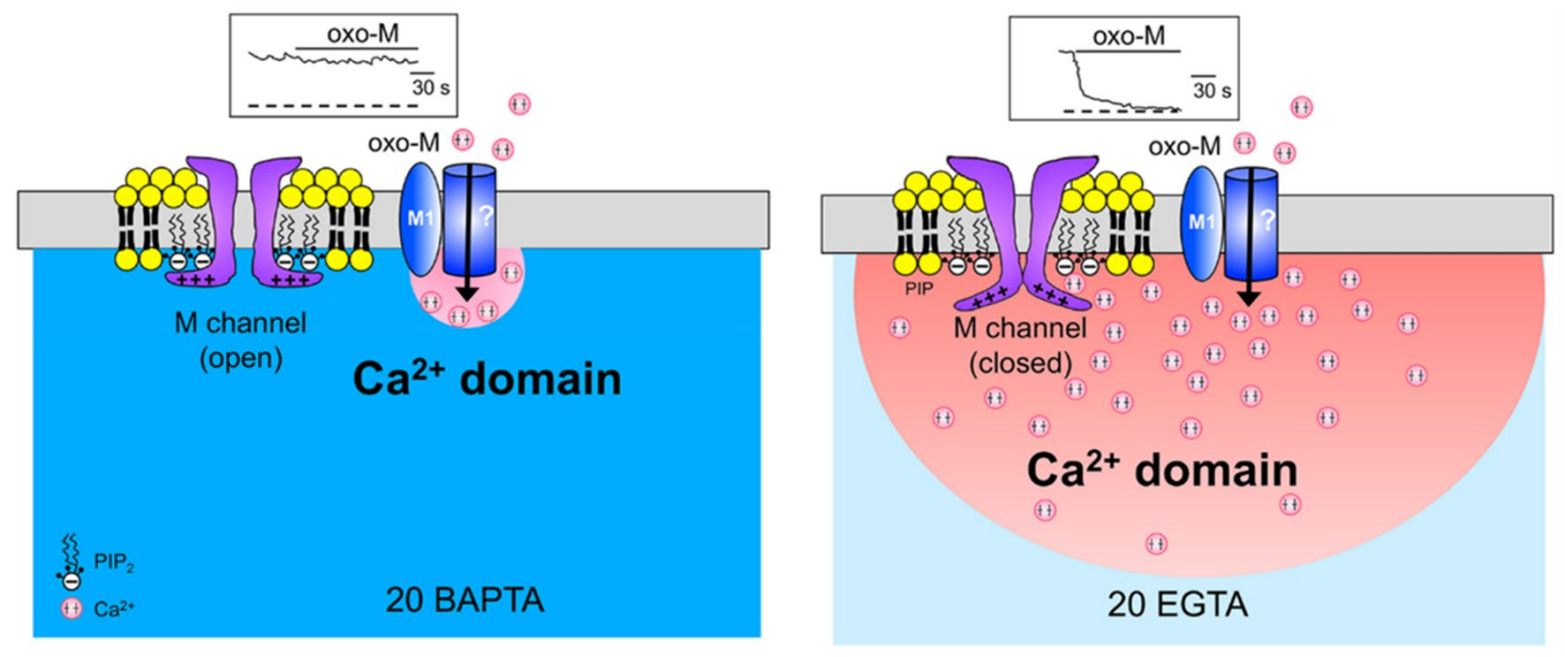
Model for Ca^2+^ microdomains for muscarinic modulation of I_M_ in SCG neurons. The length constant of Ca^2+^ microdomains associated with individual M1 muscarinic receptors (M1) is calculated to be ~ 5 nm and 60 nm in the presence of 20-mM BAPTA (*left*) and 20-mM EGTA (*right*), respectively. Ca^2+^ rises in response to muscarinic stimulation can lead to weakening channel–PIP_2_ interaction, resulting in the decrease of M currents

In fact, it was also observed in earlier studies that high concentrations of Ca^2+^ chelator (20-mM BAPTA) can suppress muscarinic inhibition of I_M_ (Beech et al. 1991; Kirkwood et al. 1991). However, when 10-mM Ca^2+^ was added to 20-mM BAPTA-containing pipette solutions to increase free Ca^2+^ concentration from 12 to 143 nM, oxo-M was still capable of inhibiting I_M_ (Beech et al. 1991; Cruzblanca et al. 1998). These results were interpreted to mean that the effect of BAPTA to inhibit a muscarinic effect on I_M_ was attributable to lowering of resting [Ca^2+^]_i_ rather than inhibiting the rise of [Ca^2+^]_i_ by muscarinic stimulation (Beech et al. 1991). This interpretation was based on the assumption that adding 10-mM Ca^2+^ to 20-mM BAPTA-containing solution increases free Ca^2+^ concentration without affecting Ca^2+^ buffering power during the event of rising [Ca^2+^]. That is not the case, but Ca^2+^ buffering power is compromised because BAPTA is occupied by the added Ca^2+^, resulting in reduction of free BAPTA that is capable of buffering Ca^2+^ rise. Therefore, these results and our results may present evidence that Ca^2+^ signals that mediate muscarinic inhibition of I_M_ are only suppressed by a very high concentration of BAPTA. The requirement of high concentrations of BAPTA may imply two possibilities: muscarinic inhibition of I_M_ somehow requires minimum resting Ca^2+^, as was suggested previously (Beech et al. 1991), or muscarinic inhibition of I_M_ is mediated by local Ca^2+^ signals that can be suppressed by high concentrations of fast Ca^2+^ buffer, BAPTA. To distinguish these two possibilities, we tested 20-mM EGTA, which has similar equilibrium affinities for binding Ca^2+^ with BAPTA, but its Ca^2+^-binding rate is too slow to suppress dynamic Ca^2+^ rises. Since it is expected that resting Ca^2+^ concentrations should be lowered to the same extent by EGTA as it is by BAPTA, differences in the ability of BAPTA and EGTA to block Ca^2+^-triggered responses can be regarded to represent the involvement of local Ca^2+^ signals. Indeed, we found that muscarinic inhibition of I_M_ was potently blocked by BAPTA but completely insensitive to EGTA (Fig. 5), implying that the spatial proximity between the Ca^2+^ source of oxo-M-induced Ca^2+^ rise and Ca^2+^-sensitive target proteins that mediate I_M_ inhibition is so close that the coupling can only be blocked by very high concentrations of fast Ca^2+^ buffer. We calculated the length constant of Ca^2+^ microdomain in the presence of either BAPTA or EGTA, λ, which represents the mean distance that a Ca^2+^ ion diffuses before it is captured by a buffer molecule (Fig. 6). According to equation (7) by Neher (Neher 1998), λ = √(D_Ca_/*k*_on_ [B]^o^ (*k*_on_ and [B]^o^ representing the rate constant of Ca^2+^ binding to the buffer and the concentration of free buffer, respectively). We referred diffusion coefficient of free Ca^2+^ ion (223 μm^2^/s) as reported by Allbritton et al. (Allbritton et al. 1992), and the values for *k*_on_ and the rate constant of Ca^2+^ binding to the buffer, to the study by Naraghi (Naraghi 1997), in which *k*_on_ for BAPTA and EGTA is 4.5 × 10^8^/M.s and 2.7 × 10^6^/M.s, respectively. λ is calculated to be about 5 nm and 60 nm in the presence of 20-mM BAPTA and 20-mM EGTA, respectively. Thus, our results imply that M channels are localized in the vicinity of the muscarinic receptor sensitive Ca^2+^ source within 60 nm, but away from this source at more than 5 nm. To prove this model, it is necessary to identify the Ca^2+^ source and Ca^2+^-sensitive target proteins involved in I_M_ inhibition. Further studies will be required to clarify this issue.

In addition, our results showed that muscarinic inhibition of M channel was not attenuated by blocking PIP_2_ depletion with supply of PIP_2_ (up to 200 μM), or by blocking PKC activation with a specific blocker, calphostin C, or by blocking both PIP_2_ and PKC pathways. As other potential signaling pathways may have been involved, we examined whether G_βγ_ subunits of G protein or trafficking of channel proteins were involved in muscarinic inhibition of I_M_. The results showed that neither seemed to have a critical role.

Our data demonstrate that PIP_2_ depletion is not the ‘principal’ contributor to the inhibition of I_M_ by muscarinic signaling, in contrast to the PIP_2_ depletion hypothesis for muscarinic action. Clearly, KCNQ channels are PIP_2_ sensitive: they are directly activated by exogenous PIP_2_ (Zhang et al. 2003). However, two distinct questions must be answered to evaluate the role of PIP_2_ signals. The first is whether PIP_2_ functions as a regulator of channel activity and a second, distinct question is whether PIP_2_ signals play a role in M channel modulation by muscarinic receptors. The PIP_2_ depletion hypothesis is based on a number of experiments focusing on the first question. For example, selective depletion of PIP_2_ using an engineered chemical dimerization system almost completely suppressed the current, whereas PIP_2_ directly applied to excised patches augmented the current (Suh et al. 2006; Zhang et al. 2003). These data, however, cannot rule out the possibility that during muscarinic signaling, PIP_2_ depletion plays no role in decreasing current amplitudes. To determine the role of PIP_2_ in receptor-mediated regulation of ion channels, specific experiments testing the role of PIP_2_ in receptor-induced channel modulation are needed. To do this, we and others applied exogenous PIP_2_ into neurons during G_q_PCRs signaling. This experimental protocol was used to confirm the role of PIP_2_ depletion in G_q_PCR-mediated GIRK channel regulation (Cho et al. 2005, 2001; Meyer et al. 2001). As opposed to PIP_2_-mediated GIRK channel regulation, muscarinic inhibition of KCNQ channels was not suppressed by PIP_2_ (up to 200 μM, Fig. 2) and only partially reduced even by 500-μM PIP_2_ (Robbins et al. 2006). These results underscore the notion that PIP_2_ depletion might play only a small role in muscarinic inhibition of I_M_. Another piece of evidence in favor of the PIP_2_ model is that the dynamics and extent of channel inhibition by muscarinic receptors showed a close correlation to the translocation of the PH-GFP construct that provided a measure of overall PIP_2_ hydrolysis (Hernandez et al. 2008b; Horowitz et al. 2005). However, the dynamics of PIP_2_ hydrolysis does not necessarily correlate to the dynamics of PIP_2_ depletion, especially in the proximity of a given ion channel. Since no method is available to measure changes in local concentrations of PIP_2_, we developed a two-dimensional diffusion model to estimate them. Using this model, we found that the key to determining the spatial and temporal profiles of PIP_2_ depletion is PIP_2_ mobility (Cho et al. 2005), in that a profound PIP_2_ depletion restricted to the microdomain adjacent to PLC occurs only when PIP_2_ mobility is low. However, in general, diffusion constants for PIP_2_ in neurons are not low (~ 0.5–2 μm^2^/s, (van Rheenen and Jalink 2002; Cho et al. 2005)) and there is no evidence that the physical properties of PIP_2_ in SCG neurons are dissimilar from those of PIP_2_ in other neurons. When PIP_2_ is diffusible, the PIP_2_ depletion induced by PLC activation is readily attenuated by diffusion, so that the resulting changes become slower and smaller (Cho et al. 2005; Cui et al. 2010). This is in contrast to the rapid and profound I_M_ inhibition observed during muscarinic stimulation. Support for the PIP_2_ model also comes from the fact that the recovery from inhibition requires cytoplasmic ATP and is delayed by inhibition of the PI-4-kinase, which replenishes PIP_2_ (Ford et al. 2003; Suh and Hille 2002). However, although recovery from inhibition requires resynthesis of PIP_2_, whether the process of inhibition itself results directly from PIP_2_ breakdown is unclear. It must be noted that for GIRK channels in HEK cells, the recovery from receptor-induced inhibition depends primarily on PIP_2_ regeneration but receptor-induced inhibition results primarily from the change in the channel’s affinity for PIP_2_ by other signaling molecules (Brown et al. 2005). Furthermore, as mentioned earlier, although muscarinic inhibition of I_M_ is reduced by overexpressing the synthetic enzyme PI-5-kinase (Winks et al. 2005), whether muscarinic receptor signaling no longer functions in cells with increased resting PIP_2_ level caused by overexpression of PI-5-Kinase needs to be tested and resolved.

Reportedly, Ca^2+^ elevation does not require as much G_q_PCR stimulation or local receptor density as does PIP_2_ depletion or M channel inhibition (Falkenburger et al. 2013; Dickson et al. 2013; Kruse and Whitten 2021). Thus, Ca^2+^ signaling sensitizes SCG neurons to M_1_R activation, allowing I_M_ inhibition even when M_1_R is at low receptor density or under minimal muscarinic acetylcholine stimulation.

The results of our study do not exclude or support the possibility that PIP_2_ depletion and Ca^2+^ elevation might occur simultaneously in response to muscarinic stimulation; and this could enhance the inhibitory effect. However, we do show that use of exogenous Ca^2+^ buffer to inhibit Ca^2+^ rises could block muscarinic inhibition, whereas exogenously restoring PIP_2_ levels could not, suggesting Ca^2+^ elevation, not PIP_2_ depletion, is the principal driver of muscarinic inhibition of M currents.

## Supplementary Information

Below is the link to the electronic supplementary material.

Supplementary file1 (DOCX 148 KB)

## Data Availability

The datasets generated and/or analyzed during the current study are available from the corresponding author on reasonable request.

## Abbreviations

CaM: Calmodulin
G_α_TD: G_α_-transducin
GFP: Green fluorescent protein
I_M_: M channel current
M_1_R: M1 muscarinic (acetylcholine) receptor
M channels: M-type (KCNQ/Kv7) K^+^ channels
NCS-1: Neuronal calcium sensor-1
Oxo-M: Oxotremorine-M
PI: Phosphoinositide
PIP_2_: Phosphatidylinositol 4,5-bisphosphate
PI-5-kinase: Phosphoinositide-5-kinase
PKC: Protein kinase C
PLC: Phospholipase C
SCG: Superior cervical ganglion
TTX: Tetrodotoxin
