# Supplementary material for: Involvement of Ca2+ in Signaling Mechanisms Mediating Muscarinic Inhibition of M Currents in Sympathetic Neurons
Source: Cell Mol Neurobiol. 2022 Nov 11;43(5):2257–71. doi: 10.1007/s10571-022-01303-7 (PMC10287826; doi:10.1007/s10571-022-01303-7)
Supplement: Supplementary file 1 — Supplementary file1 (DOCX 148 KB) [file 10571_2022_1303_MOESM1_ESM.docx]

**Supplementary Information**

**Article title:** Involvement of Ca^2+^ in Signaling Mechanisms Mediating Muscarinic Inhibition of M Currents in Sympathetic Neurons

**Journal name:** Cellular and Molecular Neurobiology

**Author name:** Jin-Young Yoon, and Won-Kyung Ho

**Affiliation and e-mail address of the corresponding author:** Jin-Young Yoon, PhD,

Department of Internal Medicine, Division of Cardiovascular Medicine, University of Iowa, Iowa City, Iowa, USA; Department of Physiology, Seoul National University College of Medicine, Seoul, Korea.

jinyoung-yoon@uiowa.edu

**SI 1**


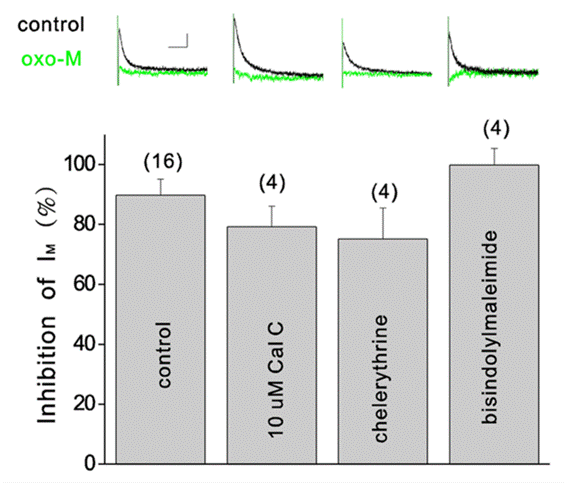


**SI 1** Effects of PKC inhibitors on muscarinic inhibition of I_M_. Superimposed current traces (upper panel) and summary data of the percent inhibitions by 10 μM oxo-M (lower panel) in control conditions or in cells with PKC inhibition ; calphostin C (10 μM, n=4), chelerythrine (1 μM, n=4), and bisindolylmaleimide I (100 nM, n=4). Values are expressed as means±S.E.M. The numbers in parentheses indicate the number of cells tested. Scale bars: 50 pA and 200 ms

**SI 2**


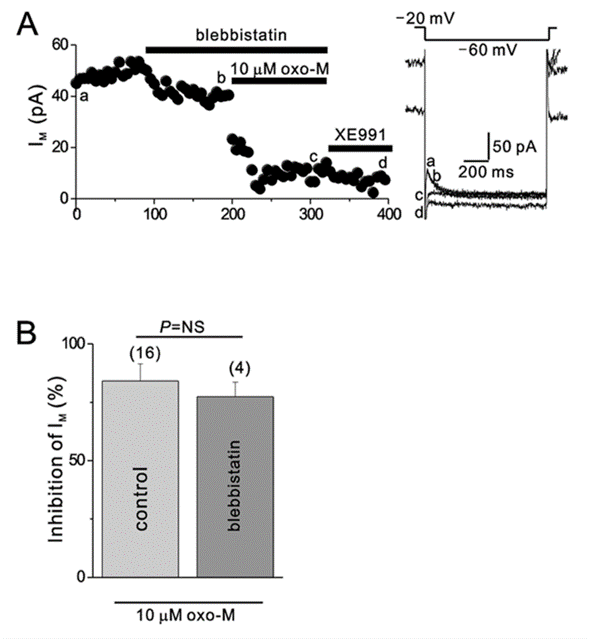


**SI 2 Blocking of protein trafficking by blebbistatin did not affect the muscarinic inhibition of I_M_.** (A) The effects of 10 μM oxo-M on I_M_ in cells which were pretreated with blebbistatin. *Inset* shows the pulse protocol and representative current traces. (B) Summary of the percent inhibitions of I_M_ by oxo-M in control conditions or in cells pretreated with blebbistatin. Values are expressed as means±S.E.M

**SI 3**


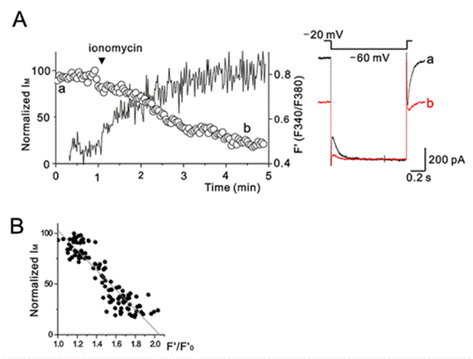


**SI 3** Ionomycin-evoked increase of [Ca^2+^]_i_ suppressed M current. (A) simultaneous recordings of Fura-2 ratios (solid line) and M current (open circles) were done in a neuron loaded with Fura-2 dye through the patch pipette. *Inset* shows the pulse protocol and representative current traces. (B) Relationship of Fura-2 ratios to the amplitude of M currents for the cell depicted in *A*. Calculation of their relationship under these conditions yielded a linear correlation coefficient of -0.84±0.02 (n=4, p<0.0001)
